# Governance and Health Aid from the Global Fund: Effects Beyond Fighting Disease

**DOI:** 10.5334/aogh.2505

**Published:** 2019-05-13

**Authors:** Matthew M. Kavanagh, Lixue Chen

**Affiliations:** 1 O’Neill Institute for National and Global Health Law, Georgetown University, US

## Abstract

**Background:**

The Global Fund to Fight AIDS, Tuberculosis and Malaria has proven highly effective at fighting the world’s major killers. Strong governance and robust development institutions are necessary, however, for improving health long-term. While some suggest that international aid can strengthen institutions, others worry that aid funding will undermine governance, creating long-term harm. The Global Fund is a unique aid institution with mechanisms designed to improve transparency and accountability, but the effectiveness of this architecture is not clear.

**Objectives:**

This study seeks evidence on the effects of Fund financing over the past 15 years on national governance and development.

**Methods:**

A unique dataset from 112 low- and middle-income countries was constructed with data from 2003 to 2017 on Global Fund financing and multiple measures of health, development, and governance. Building a set of regression models, we estimate the relationship between Fund financing and key indicators of good governance and development, controlling for multiple factors, including the effects of other aid programs and tests for reverse causality.

**Findings:**

We find that Global Fund support is associated with improved control of corruption, government accountability, political freedoms, regulatory quality, and rule of law, though association with effective policy implementation is less clear. We also find associated benefit for overall adult mortality and human development.

**Conclusion:**

Our data are not consistent with recent claims that aid undermines governance. Instead our findings support the proposition that the Global Fund architecture is making it possible to address the continuing crises of AIDS, tuberculosis, and malaria in ways that improve institutions, fight corruption, and support development. Amidst the complex political economy that produces good governance at a national level, our finding of a beneficial effect of health aid suggests important lessons for aid in other settings.

## Introduction

The Global Fund to Fight AIDS, Tuberculosis (TB) and Malaria has been widely lauded as among the most effective international aid programs, investing nearly US$4 billion a year to support programs in more than 100 countries. The Fund’s most recent report shows that in 2017 it contributed toward delivery of HIV treatment for 17.5 million people, TB treatment for 5 million, and distribution of 197 million bed nets to prevent malaria in low- and middle-income countries [1]. Millions of lives have been saved [2,3].

Support for scaling up health services, however, is only one part of what is needed for long-term success against these major killers. Countries that succeed in building effective health systems, able to combat disease and improve overall population health, rely on strong systems of governance. Where institutions of power and politics ensure wide participation, transparency, robust accountability, effective capacity to regulate, checks on corruption, and a strong legal system, governments and societies are better able to coordinate action to improve well-being [4,5,6]. These institutions not only drive health, which is itself a key macroeconomic factor, but are linked to economic strength and development overall [7].

In this context, Global Fund’s effects on governance are an important aspect of its impact in the world beyond the three diseases. While some suggest international aid efforts can improve both development and governance by improving systemic capacities, others worry aid is “dead” or a “curse” that undermines accountability and permits corruption [8,9,10]. The Global Fund is a distinctive aid institution—focused on specific health outcomes, with programs resourced through a public-private partnership. As described below, the Fund has introduced a set of innovative structures, unique to the architecture of aid, explicitly designed to improve governance and negate the distortions of aid financing.

It could be that Global Fund financing has negligible effect, for good or ill, on broader governance and thus disease-fighting efforts can be judged by themselves. On the other hand, these mechanisms could prove insufficient to the task, with Global Fund aid driving disease benefits but governance harms, in which case relative priorities would need to be considered. A third possibility is that the participation, accountability, and capacity-building architecture of the Fund have a wider beneficial impact in the countries receiving aid. To bring evidence to this debate, we describe the results of an analysis of a unique dataset on Global Fund financing alongside key indicators of governance and development over the past 15 years to empirically explore the impact of the Fund in this area.

## Aid, Governance, and Health

The political economy of foreign aid has been hotly debated, with few points of consensus. Aid optimists argue that ensuring sufficient resources in a country can, under the right circumstances, achieve the goals of increasing economic growth, mitigating poverty, and improving provision of public goods [11,12,13]. Skeptics have challenged this link and suggested aid can increase dependency while undermining development [9,14]. Governance has been a particular point of concern. Broadly defined, governance includes how governments are selected, monitored, and replaced; capacity to formulate and implement policies; and respect of citizens and the state for core rights and rules. These institutions have been shown to be critical for economic and human development [7,15]. Thus, aid that achieves goals like expanding access to services but undermines institutions could be a development net negative. Critics worry that funding public goods through aid, rather than from citizens and taxation, can undermine political accountability, democracy, economic regulation, and institutional controls under law. Empirical evidence on this front is mixed—some find a net-negative effect of aid on governance, while studies using different models find overall benefit [10,16,17,18,19]. Corruption has been a particular focus and, while Okada and Samreth find aid decreases corruption as did Tavares, Asongu and Nwachukwu find the opposite [20,21,22]. Treating all aid as similar, however, is a mistake and may explain conflicting evidence. Most “official development assistance” (ODA) is based on the political objectives of the donor countries, while only a small portion of aid is targeted based on the needs of lower-income recipient countries [23].

Global health aid, in this context, is the exception. Empirical studies have shown that health-specific aid *does* tend to have the intended effect on improving well-being [24,25,26,27]. Health aid is also more often allocated based on need than broader ODA, although not without the influence of donor preferences [28,29]. The effects of health aid on governance, however, have been under-explored. Addressing governance is particularly important for health, with ample evidence that good governance and controlling corruption increase the effectiveness of health spending from both domestic and international sources [26,30]. In this context, understanding the effects of Global Fund financing in these areas has important implications.

## The Global Fund and Governance

**Figure 1:**
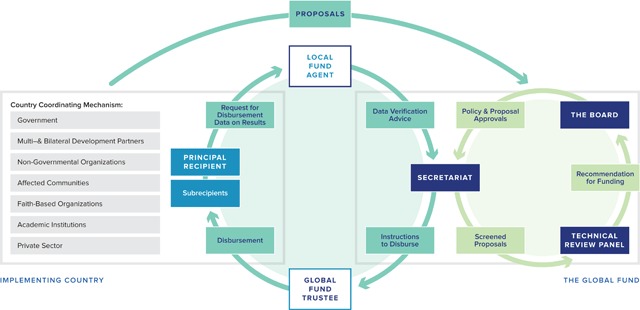
Global Fund Architecture. **Source:** The Global Fund Strategy 2017–2022: Investing to End Epidemics.

The Global Fund was created in 2002 at the height of the AIDS pandemic as a novel institution—a global health financing mechanism that pools voluntary contributions from wealthy nations, foundations, and the private sector for distribution to both public-sector and non-governmental implementing agencies. Governance was a particular concern from the start, with several key innovations developed to address internationally recognized weaknesses of aid (See Figure 1). All financing is required to be based on a plan developed by the country for the overall response to AIDS, tuberculosis, and/or malaria. A Country Coordinating Mechanism (CCM) at country level prepares proposals to the Fund and oversees implementation—with representation required from government, non-governmental organizations (NGOs), civil society, multilateral and bilateral agencies, and the private sector. Each grant (countries may have multiple streams of funding) has a principle recipient (PR) organization that receives funds and is responsible for implementation and monitoring. These PRs may be government agencies (e.g., Ministry of Health), NGOs (e.g., Action Aid in Malawi) or international organizations (e.g., United Nations Development Program) and are responsible to the CCM. An independent professional local fund agent (LFA) is contracted in each country to provide oversight and verification of financial and programmatic progress. At the global level, each application is reviewed by an independent technical review panel of experts that makes recommendations to the board of the Fund, which has the ultimate say over all expenditures. That board includes seats for donor countries and recipient (implementing) countries, private foundations, NGOs from both the global North and South, communities affected by the three diseases, and the private sector. Over time, funding mechanisms have evolved, most recently in 2013, when a “new funding model” streamlined the proposal process while increasing requirements for cross-sectoral dialogue and participation.

These unique structures were set up explicitly to address governance challenges in aid. The CCM and board are intended to address transparency, participation, and representation at country and global level. Efforts to check corruption come through the CCM, LFA, and activities by the Global Fund secretariat and inspector general—each of which have repeatedly identified and exposed misallocation of funds by public and private actors. Working through local courts and legal systems, the Global Fund has recovered large portions of misallocated or misappropriated funds [31]. The Fund has also invested significantly in human rights programming intended to strengthen rule of law [32]. Funding has flowed to implementing organizations and governments to support strengthening financial and program management systems.

The net effect of these structures and programs is not fully clear. The Global Fund’s role here is important, because its long-term health impact beyond scaling up disease programs hinges, in part, on whether its financing has a net neutral, positive, or negative effect on key aspects of governance.

## Methods and Indicators

In order to estimate the impact of Global Fund financing on governance and development, we assembled a unique panel dataset of funds distributed by country per year from 2003 to 2017 and modeled the effect of that financing. As indicators of governance, we used data developed for the Worldwide Governance Indicators (WGI) project [33], which reports data for over 200 countries across multiple dimensions of governance. While all available indicators of governance are imperfect and have faced critique, the WGI data are widely used and have been validated through multiple efforts [34,35].

The concept of “governance” has multiple inter-related aspects that might theoretically be affected by Global Fund aid. The distinct elements explored here are listed in Table 1. Control of corruption is a key basic component—reflecting whether public assets are properly used for public benefit and whether people are free from having to navigate bribes and exploitation from government officials [36]. Corruption in health systems is a significant problem, with one study finding that, in 42 out of 109 countries surveyed, more than half of citizens believed that the public health sector was corrupt or very corrupt [37]. The indicator of “voice” and accountability captures alignment with core democratic rights and political freedoms, which allow people in a country to share and receive information, organize, and ensure government is answerable to the public. These are directly associated with health and development, because open, transparent, and accountable governments are better able to respond to the needs of people and both share and receive information to improve well-being [38]. Regulatory quality reflects the degree to which governments can properly implement policies to govern non-public actors and sectors of life ranging from the workplace to the home. Weak governments often lack the capacity to do so, including on issues critical for health, safety, and welfare. Government effectiveness is among the most nebulous aspects of governance, seeking to capture a range of concepts including the quality of public infrastructure, whether civil servants are able to do their job without political pressure, and whether they adopt “high quality” policies and then properly implement them without overly frequent fluctuation. Rule of law, meanwhile, reflects another inter-related aspect of governance primarily related to whether courts and law enforcement are respected and function well—which is critical for a well-functioning health system and broader economy. These distinct measures might each be affected by health aid, for good or for ill. Moving beyond governance, the Human Development Index (HDI), created by the United Nations Development Program based on the work of Amartya Sen, measures the relative “development” of a nation based on the well-being of its people and its economy. In our data, it interacts with other controls and should be interpreted cautiously but nonetheless provides a useful, widely agreed-upon measure. Finally, we used the basic indicator of overall adult mortality rate to measure health effects that are larger than the three diseases.

**Table 1:** Health & Development Indicators.

Governance (Source: Worldwide Governance Indicators, 2018)	

**Voice and accountability**	The extent to which a country’s citizens are able to participate in selecting their government, as well as freedom of expression, freedom of association, and a free media.
**Government effectiveness**	The quality of public services, the civil service and the degree of its independence from political pressures, the quality of policy formulation and implementation, and the credibility of the government’s commitment to such policies.
**Regulatory quality**	The ability of the government to formulate and implement sound policies and regulations that permit and promote private sector development.
**Rule of law**	The extent to which agents have confidence in and abide by the rules of society, and in particular the quality of contract enforcement, property rights, the police, and the courts, as well as the likelihood of crime and violence.
**Control of corruption**	The extent to which public power is exercised for private gain, including both petty and grand forms of corruption, as well as “capture” of the state by elites and private interests.
**Development & Health**	

**The Human Development Index (HDI)**	Summary measure of average achievement in key dimensions of human development: a long and healthy life, being knowledgeable and have a decent standard of living. *Source:* UNDP, 2018
**Adult Mortality Rate (per 1,000)**	The probability of dying between the ages of 15 and 60—that is, the probability of a 15-year-old dying before reaching age 60, if subject to age-specific mortality rates of the specified year between those ages. *Source:* World Bank, 2018

Global Fund spending, our main input of interest, is based on data obtained from the Global Fund Secretariat, scaled to thousands of US dollars. The question explored here is whether governance and development are affected when greater financing is provided to fight disease through the unique mechanisms of the Global Fund. We used two different conceptualizations of what constitutes *more* funding, both used in the literature—*total* financing or financing per capita—in different models. We believe that total expenditure is the best indicator, because a $1 million program and a $100 million program are likely to have a qualitatively different impact on institutions, whether in a country the size of Nigeria or the size of Malawi. On the other hand, it might be argued that, for some aspects of governance like voice, impact might be relative to the size of the population, so we also tested per capita spending.

We included a number of control variables in order to address alternative explanations. Source and scaling for all control variables are included in Appendix 1. Wealthier countries generally score better on measures of governance, given increased capacity across multiple fronts. We therefore controlled for income level in our models—operationalized as both a categorical variable based on World Bank classification (used in main regression) and as gross domestic product per capita (used in robustness checks without significant change to our findings). We also noted the clear link between governance and war, violence, and political instability. This is particularly relevant for our inquiry, because this is likely a confounder in our data, with evidence that Global Fund financing flows at lower levels to countries in conflict [39]. We therefore included the WGI indicator for violence and instability as a control variable. Because governance and development are strongly influenced by time trends, we added a control for time by including year of observation as a variable.

To disentangle the effect of the Global Fund from that of other aid programs, we also controlled in models 2 and 3 for total aid, operationalized as the total official bilateral development assistance received by a country per capita from all Organisation for Economic Co-operation and Development countries. This includes funding, for example, from the US President’s Emergency Plan for AIDS Relief, among many other bilateral programs.

Reverse causality is a key alternative explanation that must be considered. That is, it may be that better governance drives increased aid from the Global Fund, rather than vice versa. First, it is important to note that Fund policy does not formally take account of governance when allocating funds. Hundreds of millions have been invested, for example, in Botswana, with control of corruption in the 79th percentile globally, and in Chad, which falls in the 5th percentile [33]. It is possible, though, that allocation decisions are subtly influenced by perceptions of governance triggering a reverse causality. Numerous empirical studies have explored whether better governance drives more aid, though results are mixed [40,41,42]. Fielding studies health aid specifically and shows that overall, governance has no impact on allocation, but that countries with better control of corruption do receive more health aid [28]. To account for this, we took two steps: First, as discussed above, we included overall aid levels as a control, which should account for much of the impact if governance is driving aid allocation. In addition, we included a country’s control of corruption score at the time of the first Fund grants in 2003 as a control in our full model.

Using these variables, we estimated the following as our main complete model (model 5):

\begin{array}{l}
{{\bf{y}}_{\bf{it}}} = {{\boldsymbol{\beta}}_{{\bf{0t}}}} {\boldsymbol +} {{\boldsymbol{\beta}}_{\bf{1}}}\ {\bf{Global}}\ {\bf{Fund}}\ {\bf{financing}}_{\bf{i,\, t-1}}\\
\qquad\ {\boldsymbol +} {{\boldsymbol{\beta}} _{{\bf{2}}}}\ {\bf{national}}\ {\bf{income}}_{\bf{i,\, t-1}} \\
\qquad\ {\boldsymbol +} {{\boldsymbol{\beta}} _{\bf{3}}}\ {\bf{political}}\ {\bf{stability}}{/}{\bf{violence}}_{\bf{i,\, t-1}}\\
\qquad\ {\boldsymbol +} {{\boldsymbol{\beta}} _{{\bf{4}}}}\ {\bf{other}}\ {\bf{aid}}_{\bf{i,\, t-1}}\\
\qquad\ {\boldsymbol +} {{\boldsymbol{\beta}} _{\bf{5}}}\ {\bf{baseline}}\ {\bf{corruption}}_{\bf{i}} {\boldsymbol +} {\boldsymbol t} {\boldsymbol +} {\boldsymbol{\alpha}} {\boldsymbol +} {\boldsymbol{\mu}}_{{\bf{it}}} {\boldsymbol +} {\boldsymbol{\varepsilon} _{{\bf{it}}}}
\end {array}

Y represents governance and development outcomes. Here, ***t*** represents time trends controlling for year of observation, and **μ** and **ε** are the error terms between and within country, respectively.

A wide variety of estimation strategies are available for time series, cross-sectional panel data—none of which are perfect. This article employs ordinary least squares, widely used because of its ease of interpretation and strong efficiency, with independent variables lagged by a year (Table 3). Since time series, cross-national data of the type we have often display contemporaneous correlation across units and unit level heteroskedasity, we follow the suggestion of Beck and Katz to use panel corrected standard errors to allow for better causal inference [43,44]. As Reed and Ye find, “estimators that perform well on efficiency grounds may perform poorly when estimating confidence intervals, and vice versa” [45]. We therefore also tested our main models using Parks’ Feasible Generalized Least Squares (FGLS) estimator, a common alternative to ordinary least squares, though it can underestimate error terms in comparatively small and finite samples like those used in cross-national research [46]. It does not change the substance of our findings. In our main regressions we report results from a random effects model to capture both within and between country effects, with time (year) as a continuous variable to avoid biasing the estimate of slow-changing variables like governance [47]. While some have argued for using fixed effects to deal with omitted variable bias, this does not fit our theory (which includes between-country effects) well, and the inclusion of unit dummies severely biases the estimates of the true effect of slowly changing dependent variables such as ours [47].

We performed a series of additional robustness checks on our results. Since our theoretical question suggests effects within and between countries, we used a random effects model. A Hausman test was applied, showing this is not inferior to other models. We also tested whether Global Fund financing is simply acting as a proxy for disease prevalence by including measures of prevalence in our models, which do not change our findings.

## Results

We find evidence of a significant, beneficial effect of Global Fund aid on governance and development. Across multiple model specifications, increased Global Fund financing is associated with better control of corruption, regulatory quality, voice and accountability, and rule of law. The link between financing and government effectiveness was significant only in one specification. Improved overall health and development as measured by HDI and the total adult mortality rate are also shown, across all specifications, to be associated with increased investment by the Global Fund.

Table 2 shows a summary of the models indicating whether the effect of financing was beneficial (positive governance and HDI, lower adult mortality) *and* statistically significant at least at the 10% level and, in most cases, at the 5% and 1% level. Table 3 below and Appendix 2 provide the full regression output tables. The coefficients for spending in all models were small. This is as expected, since health aid plays only a small part in the overall political economy of a nation that produces the institutions of governance measured in these indicators. We first modelled the effect of total Global Fund financing while controlling for national income, stability and violence, and time trends (model 1). We then added bilateral aid in model 2. Both models show significant beneficial effect on all variables of interest except government effectiveness. We tested whether shifting to measuring Global Fund financing on a per capita basis matters in a simple bi-variate with no controls (model 3) and with most controls added (model 4). In the former, government effectiveness gains significance; in the latter, voice and accountability loses significance; both for the only time in the model, while all others remain consistent. Finally, in our full model, we added baseline corruption to control for the possibility of reverse causality where countries with better corruption scores (the only significant predictor of aid in Fielding’s study [28]) *ex-ante* simply got more investment. As seen in Table 3, baseline corruption is a significant predictor, but Global Fund financing maintains its significance and impact. In a robustness check, we found baseline corruption is not a significant predictor of Global Fund investment, adding support to a causal effect of Global Fund funding on governance.

**Table 2:** Effect of Global Fund Spending on Governance & Development.

Statistical Models	Control of Corruption	Regulatory Quality	Voice & Accountability	Gov’t Effectiveness	Rule of law	Human Development Index	Total Adult Mortality

	**• = Significant & Beneficial Effect** (e.g. better governance, lower mortality)						

**Model 1: GF Spending, total** Effect of Global Fund spending controlling for national income, political stability and violence, and time	•	•	•		•	•	•
**Model 2: Controlling for Other Aid** All controls from model 1 & controlling for other aid	•	•	•		•	•	•
**Model 3: GF Spending, per capita simple** No controls	•	•	•	•	•	•	•
**Model 4: GF Spending, per capita + Aid** All controls from model 1 using per capita global fund spending & controlling for other aid	•	•			•	•	•
**Model 5: Full model Controlling for Baseline Corruption** All controls from model 1 using per capita global fund spending & controlling for other aid	•	•	•		•	•	•

**Table 3:** Global Fund Expenditure Effects on Governance & Development Indicators 2003–2017.

	(1)	(2)	(3)	(4)	(5)	(6)	(7)

Model 5: Total + Controlling for Corruption in 2003							

VARIABLES	Control of Corruption	Regulatory Quality	Voice & Accountability	Gov’t Effectiveness	Rule of law	Human Development Index	Total Adult Mortality

**Global Fund Expenditure, total**	0.0016*** (0.0002)	0.0014*** (0.0002)	0.0019*** (0.0004)	**0.0002** (0.0002)	0.0023*** (0.0003)	0.00014*** (0.00001)	–0.321*** (0.036)
National Income	0.0396*** (0.0034)	0.1925*** (0.0081)	0.1126*** (0.0113)	0.2356*** (0.0115)	0.0712*** (0.0062)	0.119*** (0.008)	–60.838*** (9.397)
Political Stability & Absence of Violence	0.1010*** (0.0115)	0.1008*** (0.0088)	0.1468*** (0.0120)	0.0743*** (0.0135)	0.1649*** (0.0073)	0.1188*** (0.0084)	–60.838*** (9.39734)
Bilateral Aid	0.0007*** (0.0002)	–0.0005*** (0.0001)	0.0018*** (0.0003)	–0.0010*** (0.0002)	–0.0001 (0.0002)	0.0084*** (0.0010)	–7.7467*** (2.5293)
Corruption 2003	0.7625*** (0.0129)	0.4688*** (0.0132)	0.4910*** (0.0205)	0.6068*** (0.0105)	0.6360*** (0.0109)	–0.00001 (0.00001)	–0.05552* (0.02931)
Observations	1,368	1,368	1,368	1,368	1,368	1,341	1,268
Number of Countries	112	112	112	112	112	110	112
Year Controls	YES	YES	YES	YES	YES	YES	YES
R2	0.781	0.405	0.313	0.669	0.690	0.696	0.249

Linear model with panel corrected standard errors. SEs in parentheses. All independent variables lagged one year. *** p < 0.01, ** p < 0.05, * p < 0.1.

Two major limitations of this study due to the available data and the nature of aid could be addressed in future research. First, the Global Fund is a relatively new institution, so expenditure data are only available since 2003, which imposes limits on the nature and complexity of the analysis we are able to conduct. Meanwhile, while we were able to control for significant confounders and find strong evidence for impact here, causal identification is challenging since countries are not randomized into receiving aid. Future research could add qualitative “causal process observations” [48] through case studies and cross-national process tracing to test our findings and deepen understanding of the mechanisms through which the Global Fund acts on governance.

## Conclusion

The Global Fund to Fight AIDS, Tuberculosis and Malaria has proven highly effective at fighting the world’s major infectious killers. Some have worried, though, that aid programs, no matter how well-intended, may undermine institutions. Our data do not support these recent critiques of “dead” aid. Instead our findings are consistent with the proposition that the Global Fund architecture is supporting ambitions to address the urgent and continuing crises of AIDS, TB, and malaria while improving institutions, fighting corruption, and supporting development. The Fund has introduced several innovative structures at the country and global levels intended to ensure that financing flows through participatory processes, with levels of transparency and accountability that are unusually high among aid programs. Activists and people directly affected by the three diseases sit on the bodies that plan and oversee programming, alongside government and technical experts. Projects are regularly audited by an independent entity. Procurement processes and regulation of the health sector are supported through technical assistance and financing to hire professionals to properly staff government agencies. Significant investments are made in human rights efforts and in building the capacity of local institutions. At minimum, these elements should help address negative properties of aid as a modality of financing health compared to taxation in a democratic system. Our data, though, are consistent with effects that go well beyond disease to improve governance. The effects are small, but significant, and include improvements in political freedoms, government accountability, quality of public services and independence of state institutions, rule of law, and controls on corruption. We did not find consistent impact on government effectiveness—an indicator focused on policy implementation, bureaucracy, and public infrastructure. This actually adds to our confidence in these findings, however, because the mechanisms described here for Global Fund impact are largely focused on participation and accountability. While these link directly to, for example, control of corruption or rule of law, they are not conceptually connected to improving bureaucratic efficiency or public infrastructure per se. Our analysis also shows that increased Global Fund financing is linked to overall better adult mortality and Human Development Index score, two linked measures of overall national development.

This analysis presents the first cross-national empirical evidence on the effects of health aid from the Global Fund on governance and development and suggests both an opportunity for expanded investment and for further research to test these findings and understand the mechanisms at work. Amidst the complex political economy that produces good governance at a national level, our finding of a beneficial effect of health aid could suggest important lessons for aid in other contexts. The Global Fund should consider putting even greater emphasis on maximizing the effectiveness of its participation, transparency, and accountability mechanisms. Recent moves to strengthen CCMs and domestic accountability could yield significant positive benefits. Meanwhile, other institutions in health and development might take lessons from the Global Fund’s focus and architecture for improving aid governance.

## Additional Files

The additional files for this article can be found as follows:

10.5334/aogh.2505.s1 Appendix 1. Control variables, scaling and source.

10.5334/aogh.2505.s2 Appendix 2. Global Fund Expenditure Effects on Governance & Development Indicators 2003–2017.
