# Supplementary material for: Governance and Health Aid from the Global Fund: Effects Beyond Fighting Disease
Source: Ann Glob Health. 2019 May 13;85(1):69. doi: 10.5334/aogh.2505 (PMC6634388; doi:10.5334/aogh.2505)
Supplement: Appendix 1. — Control variables, scaling and source. [file agh-85-1-2505-s1.pdf]

Appendix 1: Control variables, scaling and source	
**Variable & Scaling**	**Source**
Global Fund Expenditure<br>Scaled in Thousands of US dollars.	Global Fund Secretariat, per request, 2019
National Income<br>Scaled 1-4 based on low, lower-middle, upper-middle, and high income	World Development Indicators, 2018
Political Stability & Absence of Violence<br>Scaled -2.5 to +2.5	Worldwide Governance Indicators, 2018
Bilateral Aid<br>Net Official Development Assistance Received. Scaled in Thousands of Current US$	World Development Indicators, 2018
Control of Corruption in 2003<br>Scaled -2.5 to +2.5	Worldwide Governance Indicators, 2018
