# Supplementary material for: Governance and Health Aid from the Global Fund: Effects Beyond Fighting Disease
Source: Ann Glob Health. 2019 May 13;85(1):69. doi: 10.5334/aogh.2505 (PMC6634388; doi:10.5334/aogh.2505)
Supplement: Appendix 2. — Global Fund Expenditure Effects on Governance & Development Indicators 2003–2017. [file agh-85-1-2505-s2.pdf]

Global Fund Expenditure Effects on Governance & Development Indicators 2003-2017

VARIABLES	Model 1: GF Spending-Total							Model 2: Controlling for AID						
	Control of Corruption	Regulatory Quality	Voice & Accountability	Gov't Effectiveness	Rule of law	Human Development Index	Total Adult Mortality	Control of Corruption	Regulatory Quality	Voice & Accountability	Gov't Effectiveness	Rule of law	Human Development Index	Total Adult Mortality
**Global Fund Expenditure, total**	**0.0014*** **	**0.0016*** **	**0.0014*** **	**0.0002**	**0.0023*** **	**0.0001*** **	**-0.3574** **	**0.0016*** **	**0.0015*** **	**0.0019*** **	**0.0001**	**0.0024*** **	**0.0001*** **	**-0.3235*** **
	(0.0002)	(0.0002)	(0.0003)	(0.0001)	(0.0003)	(0.0000)	(0.1481)	(0.0003)	(0.0002)	(0.0004)	(0.0000)	(0.0003)	(0.000)	(0.036)
**Global Fund Expenditure, per capita**														
National Income	0.1661***	0.3092***	0.2074***	0.3599***	0.1941***	0.1270***	-62.3807***	0.1679***	0.2758***	0.2027***	0.3830***	0.1813***	0.1246***	-64.2881***
	(0.0113)	(0.0100)	(0.0152)	(0.0102)	(0.0044)	(0.007)	(7.954)	(0.0085)	(0.0096)	(0.0125)	(0.046)	(0.0043)	(0.007)	(8.295)
Political Stability & Absence of Violence	0.3446***	0.2540***	0.3278***	0.2584***	0.3677***	0.0082***	-7.6043***	0.3302***	0.2449***	0.2972***	0.1707***	0.3588***	0.0082***	-7.6270***
	(0.0142)	(0.0086)	(0.0165)	(0.0120)	(0.0107)	(0.001)	(2.415)	(0.0136)	(0.0077)	(0.0160)	(0.013)	(0.0102)	(0.001)	(2.456)
Bilateral Aid								0.0009***	-0.0003***	0.0020***	0.0001	0.0001	-0.0000	-0.0576**
								(0.0002)	(0.0001)	(0.0003)	(0.000)	(0.0001)	(0.000)	(0.028)
Corruption2003														
Observations	1,448	1,448	1,448	1,448	1,448	1,421	1,341	1,396	1,396	1,396	1,396	1,396	1,369	1,293
Number of Countries	119	119	119	119	119	117	119	115	115	115	115	115	113	115
Year Controls	YES	YES	YES	YES	YES	YES	YES	YES	YES	YES	YES	YES	YES	YES
R^2	0.410	0.339	0.241	0.484	0.458	0.720	0.325	0.401	0.306	0.236	0.469	0.439	0.708	0.325

Standard errors in parentheses.
All independent variables lagged one year.
*** p<0.01, ** p<0.05, * p<0.1

Global Fund Expenditure Effects on Governance & Development Indicators 2003-2017

	Model 3: GF Spending-per capita SIMPLE						
VARIABLES	Control of Corruption	Regulatory Quality	Voice & Accountability	Gov't Effectiveness	Rule of law	Human Development Index	Total Adult Mortality
**Global Fund Expenditure, total**							
**Global Fund Expenditure, per capita**	**0.013*** **	**0.020*** **	**0.009*** **	**0.006** **	**0.011*** **	**0.005*** **	**-8.035*** **
	(0.003)	(0.003)	(0.003)	(0.003)	(0.003)	(0.000)	(0.652)
National Income							
Political Stability & Absence of Violence							
Bilateral Aid							
Corruption2003							
Observations	1,443	1,443	1,443	1,443	1,443	1,416	1,337
Number of Countries	119	119	119	119	119	117	119
Year Controls	NO	NO	NO	NO	NO	NO	NO
R2	0.122	0.102	0.029	0.052	0.092	0.064	0.087

	Model 4: GF Spending-per capita + AID						
VARIABLES	Control of Corruption	Regulatory Quality	Voice & Accountability	Gov't Effectiveness	Rule of law	Human Development Index	Total Adult Mortality
**Global Fund Expenditure, total**							
**Global Fund Expenditure, per capita**	**0.010*** **	**0.013*** **	**0.004**	**0.001**	**0.004* **	**0.001*** **	**-4.719*** **
	(0.003)	(0.003)	(0.003)	(0.003)	(0.003)	(0.000)	(0.581)
National Income	0.281***	0.335***	0.316***	0.382***	0.255***	0.124***	-64.072***
	(0.046)	(0.057)	(0.069)	(0.047)	(0.046)	(0.007)	(7.643)
Political Stability & Absence of Violence	0.114***	0.168***	0.091***	0.170***	0.197***	0.008***	-5.630**
	(0.012)	(0.013)	(0.014)	(0.013)	(0.011)	(0.001)	(2.485)
Bilateral Aid	0.000*	0.000	0.000	0.000	-0.000	-0.000	-0.021
	(0.000)	(0.000)	(0.000)	(0.000)	(0.000)	(0.000)	(0.029)
Corruption2003							
Observations	1,396	1,396	1,396	1,396	1,396	1,369	1,293
Number of Countries	115	115	115	115	115	113	115
Year Controls	YES	YES	YES	YES	YES	YES	YES
R2	0.304	0.284	0.162	0.430	0.375	0.699	0.240

Standard errors in parentheses.
All independent variables lagged one year.
*** p<0.01, ** p<0.05, * p<0.1
